# The relationship of dietary inflammatory index and dietary patterns with premenstrual syndrome among women in Kermanshah: An analytical cross‐sectional study

**DOI:** 10.1002/fsn3.3404

**Published:** 2023-05-02

**Authors:** Samaneh Farpour, Davood Soleimani, Mehdi Moradinazar, Mehnoosh Samadi

**Affiliations:** ^1^ Student Research Committee, Department of Nutritional Sciences, School of Nutrition Sciences and Food Technology Kermanshah University of Medical Sciences Kermanshah Iran; ^2^ Research Center of Oils and Fats Kermanshah University of Medical Sciences Kermanshah Iran; ^3^ Department of Nutritional Sciences, School of Nutrition Sciences and Food Technology Kermanshah University of Medical Sciences Kermanshah Iran; ^4^ Behavioral Disease Research Center Kermanshah University of Medical Sciences Kermanshah Iran; ^5^ Research Center for Environmental Determinants of Health (RCEDH), School of Public Health Kermanshah University of Medical Sciences Kermanshah Iran

**Keywords:** dietary inflammation index, dietary pattern, premenstrual syndrome

## Abstract

Premenstrual syndrome (PMS) is a common psychological condition that occurs continuously during the luteal phase of the menstrual cycle. Potential factors in this syndrome comprise the central nervous system, hormones, genetic background, and nutritional indicators. Little is known about foods or eating patterns that may be associated with this syndrome, yet nutritional factors can be considered in strategies for the management of PMS. The current study purposed to investigate the relationship between PMS and dietary inflammation index as well as PMS and food patterns. The present cross‐sectional study was conducted on 125 women and girls aged 20–46 years who experienced symptoms of PMS. The inclusion criteria included cooperation and consent to enter the study, a body mass index of 18.5–25 kg/m^2^, no underlying disease, no use of contraceptives or antidepressants, and no use of multivitamin or mineral supplements. In the first stage of this study, participants' height, weight, waist circumference, and hip circumference were measured. In the second stage, eating habits were examined using a semiquantitative Food Frequency Questionnaire. This study found a significant correlation between glycemic load quintiles as well as between energy and macronutrient intake and the dietary inflammatory index; however, it revealed a direct correlation between PMS and both Western‐mixed dietary and high‐salt–high‐sugar dietary patterns. Moreover, the Western food pattern was found to have a direct correlation with dietary inflammatory index, and the healthy food pattern had an inverse correlation with this index. This study showed that PMS symptoms are more severe with the consumption of high‐salt–high‐sugar or a Western‐mixed food dietary pattern. It seems that an imbalance in hormones and neurotransmitters can affect the metabolism of proteins, carbohydrates, and fats. Also, some foods, such as vegetables, and low‐fat and high‐fiber diets reduce plasma estrogen levels and the duration of PMS symptoms.

## Graphical abstract

Recent studies have shown that dietary patterns may be related to and affect the symptoms of premenstrual syndrome. One symptom of this syndrome is depression, and previous studies have shown that inflammation plays a role in the pathogenesis of depression. The current study purposed to investigate the relationship between the dietary inflammation index and food patterns with premenstrual syndrome.

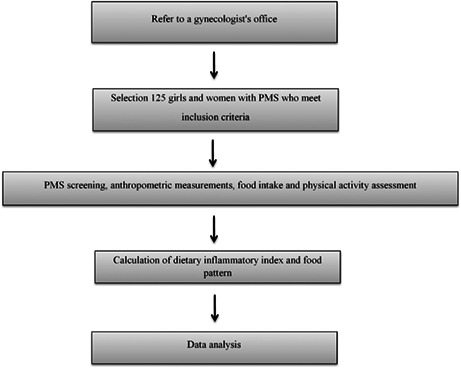

## 1 INTRODUCTION

Data have shown that 75% of women of reproductive age suffer from some symptoms of PMS; however, only 3%–8% report very severe symptoms (Gnanasambanthan & Datta, 2019). Around the world, 46% of Asia, 85% of Africa, 40% of Europe, and 60% of South America have declared the severity of premenstrual syndrome. Iran has reported a maximum prevalence rate of 98%. According to studies conducted in Tehran, Ilam, Sanandaj was more prevalent (Sahu et al., 2022). Today, women's health is a principal goal and an important tool for social and economic development. Premenstrual syndrome (PMS), a psychosomatic problem related to women's reproductive function (Pal et al., 2022), occurs at the end of the luteal phase of the menstrual cycle and affects women's daily functioning, significantly impairing their quality of life (Eshetu et al., 2022; Patel et al., 2023). Some women experience physical, behavioral, and psychological changes from a week prior to a few days into menstruation, the severity of which varies among women according to hormonal, psychological, and physiological factors (AlQuaiz et al., 2022). PMS can lead to reduced work productivity, reduced health‐related quality of life, and interference with interpersonal relationships and daily life activities. Furthermore, this syndrome may increase the risk of hypertension and reduce work‐related quality of life (Kahyaoglu Sut & Mestogullari, 2016). The cause of PMS has not yet been determined; however, dietary fats may affect hormone and cytokine levels in women. A number of retrospective studies have reported an association between PMS and fat intake (Bertone‐Johnson et al., 2014; Nagata et al., 2004). It has been shown that dietary fat and saturated fatty acid act as pro‐inflammatory agents to increase inflammatory reactive protein (CRP) (Poli et al., 2023; Turunen et al., 2013). Omega‐3 unsaturated fatty acid, as an anti‐inflammatory agent, reduces interleukin‐6 and CRP concentrations (Houghton & Bertone‐Johnson, 2015), but two theories for the etiology of PMS have been put forth. The first is that some women are sensitive to progesterone. Estrogen and progesterone levels are the same in those with and those without PMS, but studies have shown that women with PMS feel severe symptoms of the syndrome despite normal estrogen and progesterone levels. The second theory is that estrogen and progesterone reduce serotonin levels (the chemical neurotransmitter known to regulate mood). Selective serotonin reuptake inhibitors reduce PMS symptoms by increasing serotonin. EiKelis et al. found that serotonin levels were lower in obese people compared with lean people. Scientific evidence has also suggested that low levels of serotonin induce mood disorders in the premenstrual stage and lead to an increase in carbohydrate intake in the premenstrual stage in PMS sufferers (Dinh Trieu Ngo et al., 2023). Other causes involved in the etiology of PMS include inflammation and changes in hormones, among which, increased levels of CRP and other inflammatory cytokines are associated with PMS and its symptoms (Bertone‐Johnson et al., 2014; Nagata et al., 2004; Poli et al., 2023). Despite decades of research, studies have consistently failed to identify differentiating factors between PMS cases and asymptomatic women; thus, the underlying pathophysiology of PMS remains unclear. Chronic inflammation is involved in the cause of depression and other mental and physical disorders that have common characteristics with PMS. Chronic inflammation occurs when cytokine‐producing cells remain active, and it is a well‐known component of allergic and autoimmune conditions. Cytokine expression is observed in the endometrium of ovarian tissue and granulosa cells. It is believed that cytokines play a role in the recruitment of leukocytes, vessels, and tissue regeneration and repair during the menstrual cycle (Evans & Salamonsen, 2012). The results showed a negative correlation between cytokines and depression scores in the follicular phase in women with PMS symptoms, which can be interpreted as a possible mechanism of the organism to control exacerbation of the negative mood state (Foster et al., 2019). Premenstrual syndrome is associated with psychological conditions, including mood disorders and depression in particular (Fernández et al., 2018). Inflammation also plays an important role in the pathogenesis of depression (Hofmeister & Bodden, 2016). Studies have shown that when some foods increase in the daily diet, inflammation in the body also intensifies, which affects the person's mental state; the constant consumption of these foods exacerbates inflammation (Baxter et al., 2013; Vos et al., 2012). Previous research has shown that various lifestyle factors such as lack of exercise, inadequate sleep, caffeine consumption, and the consumption of unhealthy foods are reasons for PMS. In addition, progesterone affects neurotransmitters. Increased prolactin levels, insulin resistance, abnormal hypothalamic–pituitary–adrenal axis function, and nutritional deficiencies are all responsible for PMS (Sahu et al., 2022). The main objective in treating PMS is to relieve symptoms and reduce its effects on activities of daily living. The first line of treatment for PMS has been drug therapy; however, research has shown combination therapy to be the best way to relieve symptoms. A combination of pharmacotherapy (nonsteroidal anti‐inflammatory drugs, anxiolytic agents) with nonpharmacological treatments (mainly cognitive and behavioral therapy, exercise, and nutritional modification) is useful for treating premenstrual symptoms (Vaghela et al., 2019). The aim of the current study was to determine the association between PMS and the dietary inflammation index and dietary patterns among women and girls in Kermanshah, Iran. The importance of the topic and the necessity of conducting research is discussed below.

The most important parameters to consider in strategies for the management of PMS are nutritional factors; however, little information exists about specific foods and eating patterns that may be associated with PMS. Extensive research has determined that inflammation is one of the main factors in the pathogenesis of several chronic diseases, including psychological problems (Granda et al., 2021). Considering that PMS is a combination of mental and physical symptoms, we hypothesized that inflammation may play a role in the occurrence of PMS. The cross‐sectional study design was chosen for the current study, because it was conducted during the covid‐19 pandemic, and the health of the participants was a priority for the researchers. Moreover, we wanted to obtain information on the participants' dietary patterns and measure the effect these patterns have on the symptoms of premenstrual syndrome. The present study examined the association between these factors.

## 2 MATERIALS AND METHODS

The Ethics Committee (IR. KUMS. REC.1400136) of Kermanshah University of Medical Sciences approved this cross‐sectional research, and informed consent was obtained from every participant. The study population comprised 125 women and girls aged 20–46 years who were referred to a gynecologist's office in Kermanshah. Inclusion criteria were being female, willing to participate in the study, and a body mass index of 25–18.5 kg/m^2^. The average age of participants was 20–46 years. Those with underlying diseases such as diabetes, liver, and kidney dysfunction, cardiovascular disease, cancer, or polycystic ovary syndrome; those with a body mass index higher than 29.9 kg/m^2^, and those taking contraceptives, antidepressants, or multivitamin and mineral supplements were excluded from the study. A General Information Questionnaire, a Physical Activity Questionnaire, a Food Frequency Questionnaire (FFQ), and a Premenstrual Symptoms Screening Questionnaire (PSST) were used for data collection.

### 2.1 Evaluation

The questionnaires were completed in face‐to‐face interviews and by the data collection officer who was proficient in questionnaires and measurement modules. *General Information Questionnaire*: The main questionnaire gathered data on age, weight, height, body mass index, household income level, demographic, and socioeconomic information. *Physical Activity Questionnaire*: The Baecke physical activity questionnaire was used to assess the usual amount of time spent by participants on different types of physical activities. The subjects reported how much time they normally spend doing normal physical activities or exercise (light, medium, heavy) in minutes and hours (Baecke et al., 1982). The activity of the subjects was calculated based on the following formula (Morino et al., 2016):

$$\text{Physical activity}\;\left(\text{kcal}\right):1.05\times \text{body activity}\times \text{physical activity time}\;\left(h\right)\times \text{weight}\;\left(\mathrm{kg}\right).$$

*Premenstrual Syndrome Symptom Screening Questionnaire*: The Premenstrual Symptoms Screening Questionnaire (PSST) used herein consisted of two parts and a total of 19 questions. The first part consisted of 14 questions regarding moods and physical and behavioral symptoms, and the second part consisted of five questions on the effects these symptoms have on the subjects' lives. For each question, four criteria were mentioned in all, mild, moderate, and severe, which were scored from 0 to 3. Three conditions were considered to diagnose moderate or severe PMS: (1) From options 1–4, at least one was moderate or severe; (2) In addition to the previous case of options 1–14, at least four items are moderate or severe; and (3) In part, the symptoms have either moderate or severe impact on life (Hariri et al., 2013).

*Food Frequency Questionnaire*: Dietary intake was assessed through a 167‐item Food Frequency Questionnaire previously evaluated for its validity and reliability. The information gathered with the questionnaire was entered into Nutritionist Iv software to obtain an accurate measurement of each subject's intake of energy and micronutrients. To calculate the amount of energy, micronutrients, and macronutrients received, the amounts of each food mentioned in the questionnaire were converted to heat using the household scale guidelines and analyzed by (NUTRIONIST4) software (modified to suit Iranian foods) to determine the amount of macronutrients and micronutrients consumed. To calculate the glycemic index and glycemic load of the diet, the intake of food groups, macronutrients, and micronutrients was estimated using N4 software.

*Calculation of dietary inflammatory index*: The dietary inflammatory index (DII) is a scoring algorithm based on an extensive review of articles published from 1950 to 2010 that examined the association between 1943 articles as a set of dietary parameters including macronutrients and micronutrients. These parameters are rated 0 in the diet depending on whether they increase inflammation based on six inflammatory markers (IL‐1b, IL4, IL6, IL‐10, CRP, and TNF‐a). As a general dietary parameter, the specific inflammatory effect score is calculated and multiplied by a percentile‐centric value for each food. This percentile is calculated by the first association of dietary data, which is actually based on human food consumption in 11 populations from different parts of the world, which is a strong estimate of an average and standard deviation for each parameter. These values are then converted to coefficients to express a person's exposure to the global standard average of each parameter as a *Z*‐score. This is achieved by subtracting the global standard average from the reported value and dividing this value into standard deviation. This value is then converted to a percentile‐driven score. The percentile‐centered score for each food parameter is then multiplied by 2 and one was subtracted. The number obtained for each parameter is then multiplied by the inflammatory score of the parameter. Finally, all dietary parameter scores are collected to create an overall score of the DII for each study participant. The highest score on the DII that a pro‐inflammatory diet can get is −7.98, and the lowest score an anti‐inflammatory diet can get is −8.87 (Diba‐Bagtash et al., 2021). The present study calculated the inflammatory indices of the various diets using measurements of the participants' intake amounts of energy, macronutrients (carbohydrate, protein, fat), and micronutrients (sodium, potassium, vitamin A, beta‐carotene, vitamin C, calcium, iron, vitamin E, vitamin B1), vitamin B2, vitamin B3, vitamin B6, vitamin B9, vitamin B12, biotin, vitamin B5, phosphorus, iodine, magnesium, zinc, copper, manganese, selenium, fiber, garlic, caffeine, onion, monounsaturated fatty acid, and polyunsaturated fatty acids.

$$Z=\frac{\text{The value of each food intake parameter}-\text{the average global standard of each parameter}}{\text{Standard deviation}}.$$

### 2.2 Data analysis

Statistical analysis was performed using SPSS software version 19. Normal data distribution was confirmed using the Kolmogorov–Smirnov or Shapiro–Wilk test. After assessing the normality and linear correlation between the studied variables, regression and correlation analyses between the mentioned variables were evaluated using SPSS software. The significance level in all statistical tests was assumed to be .05. The study data were collected using the two methods of observation and interview. Using the available sampling method, samples were collected from gynecologists' offices from all areas of Kermanshah city, so that all strata with different economic and social levels were included in the study. The sample size was estimated using the results of previous studies (Hashim et al., 2019) and the following formula; assuming a correlation coefficient of .25 between the dietary DII with PMS and a power of 0.8 and a type 1 error of 0.05, the minimum sample size for this study was estimated to be 125 people.

## 3 RESULTS

**FIGURE 1:**
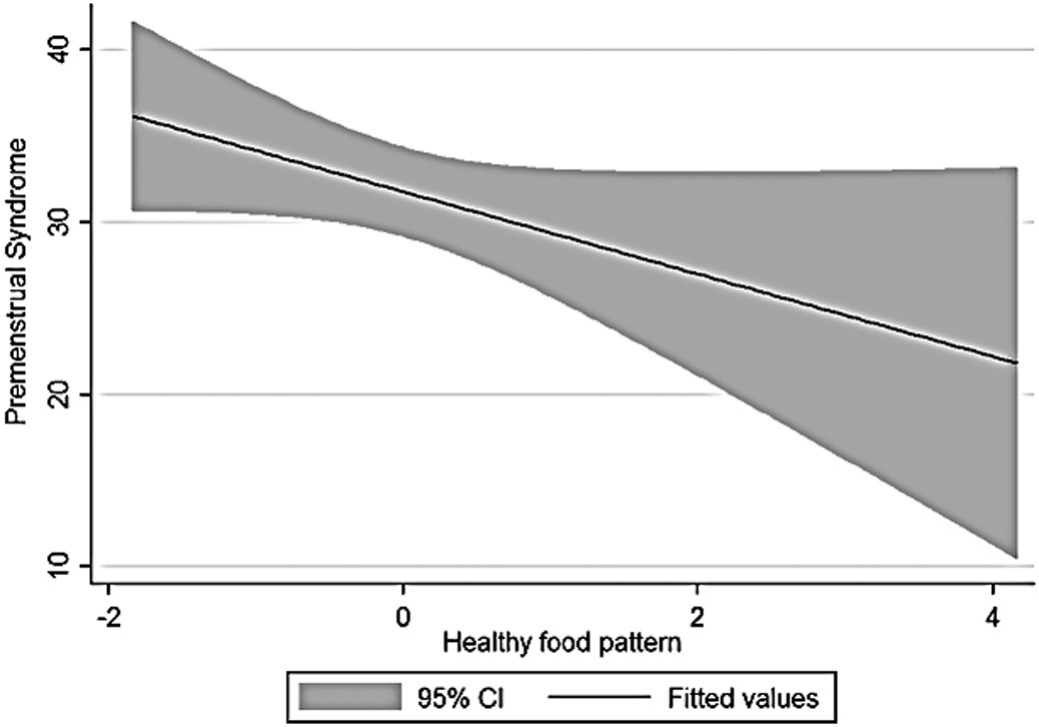
Relationship between different degrees of premenstrual syndrome and healthy food pattern.

**FIGURE 2:**
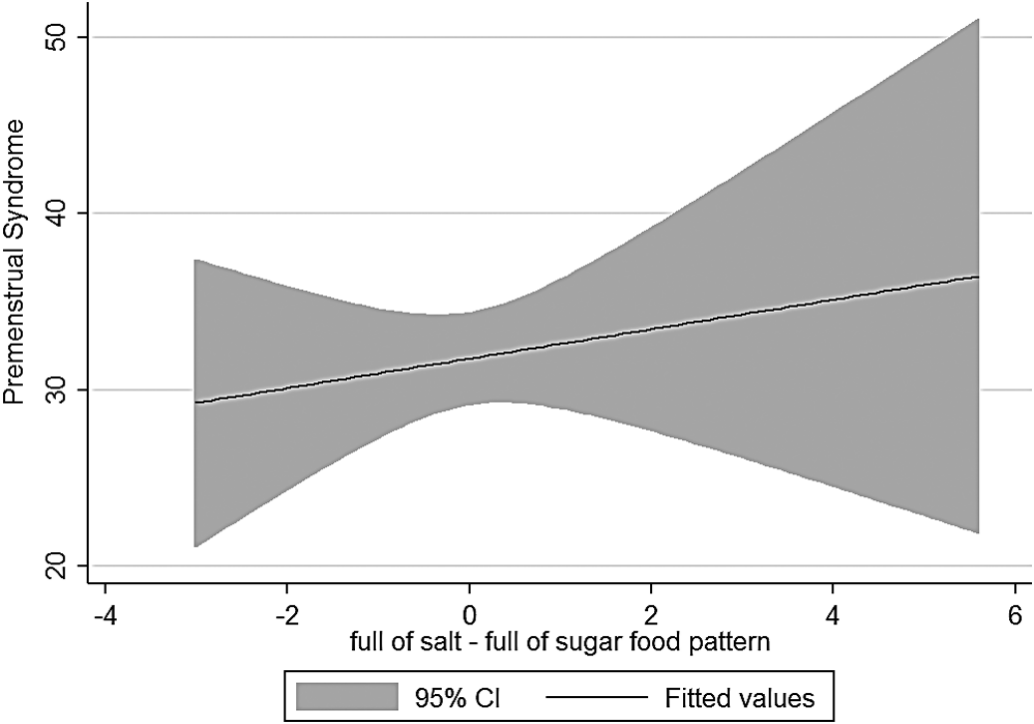
Relationship between different degrees of premenstrual syndrome and high‐salt–high‐sugar pattern.

**FIGURE 3:**
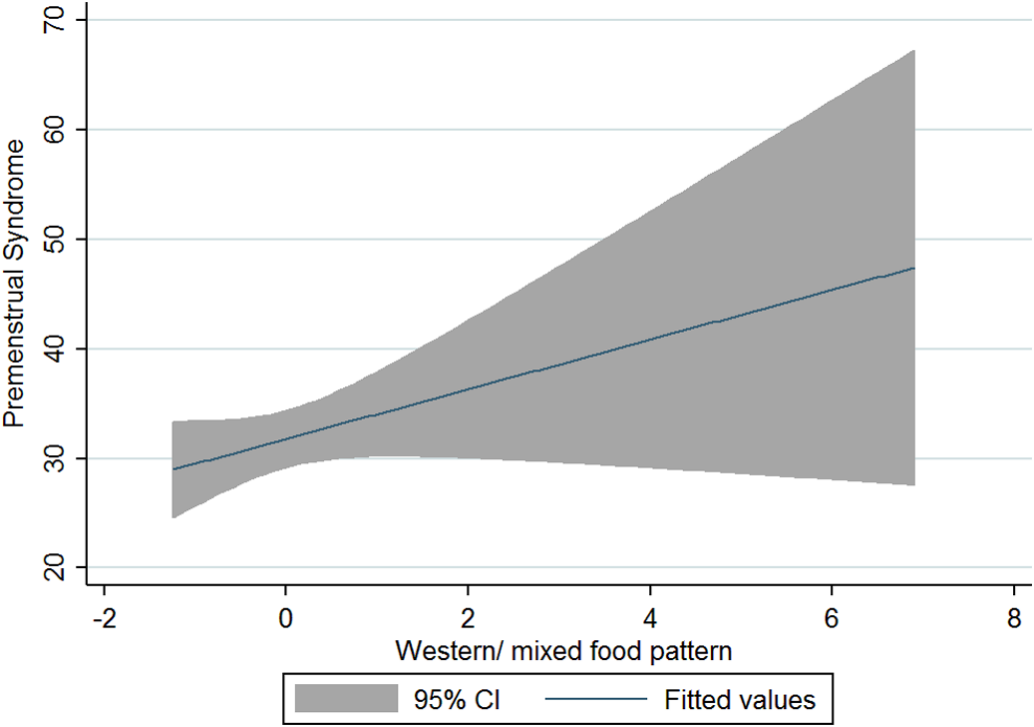
Relationship between different degrees of premenstrual syndrome and Western‐mixed food pattern.

**FIGURE 4:**
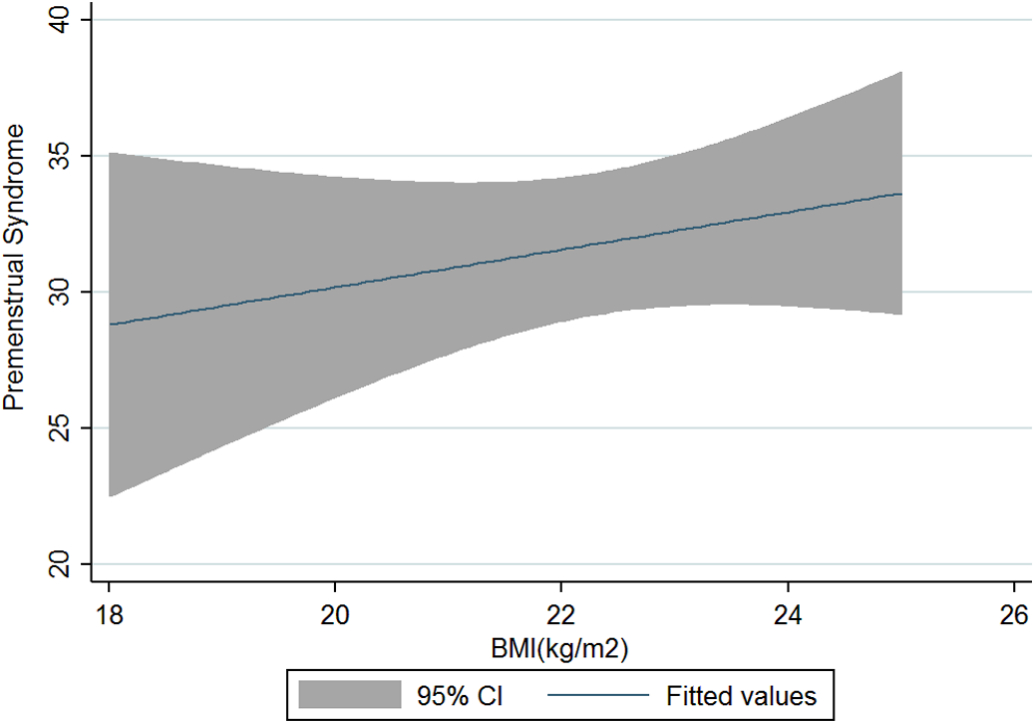
Relationship between different degrees of premenstrual syndrome and BMI.

**FIGURE 5:**
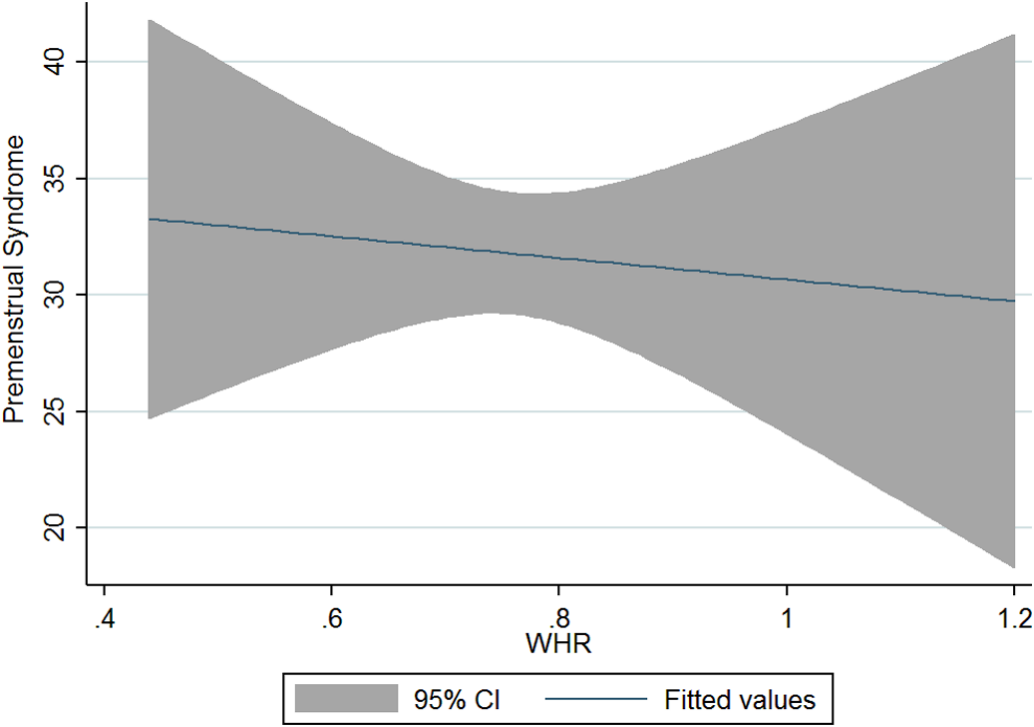
Relationship between different degrees of premenstrual syndrome and WHR.

**FIGURE 6:**
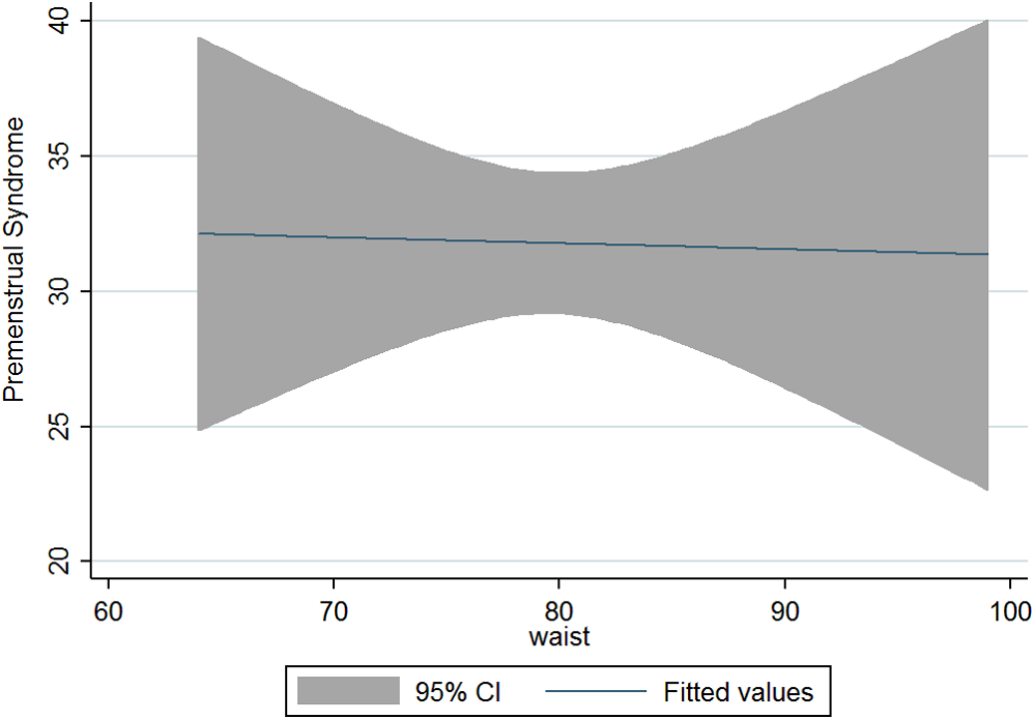
Relationship between different degrees of premenstrual syndrome and waist circumference.

The current research was conducted on 125 women and girls in Kermanshah city with an average age of 27.56 years (Table 1). The average energy consumption of the studied subjects was estimated to be 2439 kcal/day, of which 45% was provided by carbohydrates, 16% by protein, and 37% by fat (Table 2). Using the factor analysis method, three dominant food patterns (healthy, Western, and high‐salt–high‐sugar) were identified in the subjects under study. With the Kruskal–Wallis test, nutritional variables, physical activity, anthropometry, demographics based on glycemic index, and the dietary glycemic load were investigated, and the results showed that energy has a significant association with glycemic load. The association between macronutrients and DII was also significant (Table 3). Based on the results, high‐salt–high‐sugar diet and Western‐mixed diet had an effect on increasing PMS severity, and there was not any association between healthy food pattern and PMS index (Table 4, Figures 1, 2, 3). Anthropometric indices, however, did not reveal a significant association with PMS (Figures 4, 5, 6). Based on the ANOVA test, the association between PMS and the DII was significant. In the results obtained between the glycemic index, glycemic load, and premenstrual syndrome, however, no significant association was found. The association between dietary patterns and the DII was measured using the Kruskal–Wallis test, and the results revealed a significant association among all three dietary patterns and the DII (Table 5).

**TABLE 1:** General characteristics and anthropometric indicators.

Variable	Mean ± SD
Age (years)	27.56 ± 6.35
Height (cm)	163.82 ± 5.75
Weight (kg)	59.96 ± 6.62
BMI (kg/m^2^)	22.30 ± 1.93
WHR	0.76 ± 0.1
Waist/height	79.77 ± 6.03

Abbreviations: BMI, body mass index; WHR, waist–hip ratio.

**TABLE 2:** Dietary intake of the studied subjects.

Variable	Mean ± SD
Energy (kcal)	2439.53 ± 602.03
Protein (%kcal)	16.25 ± 4.77
Carbohydrates (%kcal)	45.68 ± 9.69
Fat (%kcal)	37.89 ± 9.59
Grains (serving/day)	7.23 ± 3.21
Dairy product (serving/day)	1.25 ± 1.24
Protein foods (serving/day)	9.77 ± 8.66
Fruits (serving/day)	3.36 ± 4.72
Vegetables (serving/day)	4.32 ± 3.07
Fat group (serving/day)	14.65 ± 7.88

**TABLE 3:** Comparison of demographic, nutritional, anthropometric, and physical activity variables based on DII.

Variable	DII					*p*‐Value
	1 (lowest)	2	3	4	5 (highest)	
Age (year)	27.23 ± 1.21	28.49 ± 1.37	28.78 ± 1.28	25.79 ± 0.88	27.54 ± 1.51	.49
BMI (kg/m^2^)	22.01 ± 0.38	22.13 ± 0.37	22.52 ± 0.43	22.57 ± 0.38	22.28 ± 0.37	.68
Energy (kcal/day)	2461.4 ± 106.39	2633.26 ± 97.94	2685.87 ± 111.97	1948.92 ± 131.92	2470.32 ± 100.55	.0001
CHO (%kcal)	50.72 ± 1.82	48.73 ± 1.42	40.58 ± 2.23	44.84 ± 1.83	43.2 ± 1.76	.001
Protein (%kcal)	13.28 ± 0.68	5.57 ± 6.08	16.04 ± 1.10	17.64 ± 1.15	18.76 ± 0.817	.0001
Fat (%kcal)	35.88 ± 1.82	35.42 ± 1.46	43.29 ± 2.28	37.36 ± 2.11	37.84 ± 1.56	.02
Physical activity level score	83.07 ± 7.43	90.79 ± 8.39	86.74 ± 6.94	76.08 ± 5.31	89.03 ± 6.03	.59
Level of Education	5.28 ± 0.33	5.65 ± 0.34	6.20 ± 0.21	6.12 ± 0.36	5.60 ± 0.29	.18

Abbreviation: DII, dietary inflammatory index.

**TABLE 4:** Association between premenstrual syndrome (PMS) and food patterns.

Food pattern	PMS index			*p*‐Value
	Mild (mean ± SE)	Moderate (mean ± SE)	Severe (mean ± SE)	
Healthy	0.169 ± 0.99	0.067 ± 1.017	−0.306 ± 0.81	.437
Full of salt – full of sugar	−0.145 ± 0.384	−0.001 ± 0.98	0.056 ± 0.66	.002
Western/mixed	−0.52 ± 0.459	0.012 ± 1.032	0.145 ± 0.89	.038

**TABLE 5:** Comparison of premenstrual syndrome (PMS) index based on DII, glycemic index, and glycemic load.

PMS index	DII					Total (*n*)	*p*‐Value
	1 (lowest)	2	3	4	5 (highest)		
Mild	0%	11%	22%	33%	33%	9	.536
Moderate	23%	23%	16%	19%	17%	91	
Severe	16%	16%	16%	68%	6%	25	

PMS Index	Glycemic index					Total (*n*)	*p*‐Value
	1 (Lowest)	2	3	4	5 (Highest)		
Mild	11%	22%	22%	11%	33%	9	.179
Moderate	16%	23%	21%	17%	20%	91	
Severe	36%	8%	12%	32%	12%	25	

PMS Index	Glycemic load					Total (*n*)	*p*‐Value
	1 (Lowest)	2	3	4	5 (Highest)		
Mild	0%	33%	0%	33%	33%	9	.221
Moderate	19%	16%	24%	18%	20%	91	
Severe	28%	28%	12%	20%	12%	25	

Abbreviation: DII, dietary inflammatory index.

## 4 DISCUSSION

The present study is the first analytical cross‐sectional study to investigate the association between DII and food patterns and PMS in women and girls. Using the factor analysis method, three dominant food patterns were identified in the subjects under study: a healthy food pattern (high consumption of eggs, poultry, chicken, legumes, refined grains), a salty high‐sugar food pattern (high consumption of snacks, salt, nuts, sweets, desserts, fish), and the Western food pattern (high consumption of pickles, vegetable oils, low‐fat and high‐fat dairy products, hydrogenated oil, tea, coffee, drinks). One of the most obvious findings of the current research was that the Western and salty high‐sugar food patterns affect the symptoms of premenstrual syndrome. Western and salty high‐sugar food patterns have a positive association with the inflammatory index level, while a healthy diet with high antioxidant, vitamin, and phytochemical contents leads to a decrease in the inflammatory index level. While the results of previous studies have shown that the Western diet pattern may be related to premenstrual syndrome, they did not find a significant correlation between the healthy eating pattern and PMS (Farasati et al., 2015). One of the findings of the present study is the lack of an effect of energy, macronutrient, and micronutrient intake on PMS (*p* > .05). In line with our study, Jafari et al. (2020) studied 200 women with symptoms of PMS and showed that taking zinc supplements for 12 weeks improved the subjects' physical and mental symptoms. Ismailipour et al. conducted a study on 100 nurses with symptoms of premenstrual syndrome. Their results showed that a daily intake of whole grains is helpful in improving PMS (Esmaeilpour et al., 2019). Taheri et al. (2020) studied 217 women, and their results indicated that high intake of energy and macronutrients was significantly related to PMS, which is not consistent with the current results. In the current study, no significant correlation was seen between height, weight, body mass index, waist circumference, or hip circumference and PMS. In a study conducted on 365 students by Mohammadi et al., however, the results revealed a direct and significant correlation between PMS and anthropometric indicators. In the mentioned study, the results indicated that waist circumference and hip circumference are predictors of central obesity, and the severity of PMS increases with the increase of central fat mass (Mohammadi et al., 2013). In the present study, we investigated the association between the DII and PMS based on the ANOVA test. The results revealed a significant correlation between these two variables (*p* < .05). Although the cause of PMS remains still unknown, inflammation and changes in hormones can play a role in the etiology of PMS. Dietary fats may affect the level of hormones and cytokines in women (Bertone‐Johnson et al., 2014; Nagata et al., 2004). A number of retrospective studies have reported a correlation between PMS symptoms and fat intake (Aeberli et al., 2006). It has also been shown that dietary fat and saturated fatty acid act as pro‐inflammatory agents to increase inflammatory reactive protein (CRP) (Poli et al., 2023; Turunen et al., 2013), and omega‐3 unsaturated fatty acids, as anti‐inflammatory agents, reduce the concentration of interleukin‐6 and CRP (Houghton & Bertone‐Johnson, 2015). The World Health Organization has reported depression to be the most common mental disorder. Depression is characterized by fatigue, discomfort, and a lack of interest in daily life activities (Hofmeister & Bodden, 2016). PMS is also associated with mental conditions such as mood disorders, especially depression (Fernández et al., 2018). Inflammation also plays an important role in the pathogenesis of depression (Hofmeister & Bodden, 2016). An inflammatory diet has also been found to be associated with depression (Baxter et al., 2013).

The present study showed a positive correlation between the DII and the glycemic load (*p* < .05). Studies have reported a mechanism by which a diet with a high glycemic index and high glycemic load induces inflammation. A diet with a high glycemic index and glycemic load causes hyperglycemia, which in turn induces oxidative stress (Bhatti et al., 2022). In the present study, a significant correlation was observed between energy and glycemic load (*p* < .05). The results of previous studies indicate that a diet with a high glycemic load has no effect on glucose metabolism, which is in line with the present study. A diet with a high glycemic load, however, has a negative effect on the lipid profile, but this was not found in the present study (Fernandes et al., 2022). Ismawanti et al. studied 56 type 2 diabetics, and their results indicated that an increase in blood glucose levels stimulates glycogen formation from glucose, fatty acid synthesis, and cholesterol levels from glucose, while high blood glucose levels can accelerate triglyceride formation. This study showed that a diet with a low glycemic index and glycemic load has an effect on reducing triglyceride levels (Argiana et al., 2015). In the present study, the association between macronutrients and the DII was significant (*p* < .05). The results of previous studies indicate that diet plays a major and essential role in regulating chronic inflammation (George et al., 2020). An unhealthy diet that is high in fat, refined carbohydrates, and protein is associated with high levels of inflammatory factors, while a healthy diet that contains fruits, vegetables, fish, omega‐3, and fiber is associated with low levels of inflammatory markers. In the present study, no significant correlation was found between the anthropometric and the inflammatory indices (*p* > .05). Previous studies, however, have reported that a diet with a high inflammatory index is related to body mass index and waist circumference (Choi et al., 2023).

The present study had several strengths: It is the first cross‐sectional study that simultaneously measured the association between the inflammatory index of diet and PMS and that of food patterns and PMS. The results showed the current state of the DII and the need for women with symptoms of PMS to improve their diet. This study used valid Food Frequency, Physical Activity, and Premenstrual Symptoms Screening Questionnaires, and data were collected by an experienced interviewer, which reduced the possibility of information bias. The high participation rate of the participants in the current study increased the strength of the study. The current research also had some limitations. The subjects' diets were evaluated using a Food Frequency Questionnaire, which is dependent on people's memory, and people reported their food intake as less or more than reality. Another limitation was the small sample size, which led to a lack of significance in the results. It is suggested that further studies with larger sample sizes be conducted to evaluate the association between the DII and PMS as well as that of food patterns and premenstrual syndrome. In general, the results of the present study showed that the Western food pattern, high in salt and sugar, has a positive correlation with the inflammatory index level, and a healthy food pattern reduces the inflammatory index level. Moreover, a diet with a low glycemic index and low glycemic load leads to a decrease in the rate of glucose absorption by the body as a result of reduced hyperglycemia and hyper‐insulin, which can lead to a decrease in systemic inflammation.

## 5 CONCLUSION

The current results revealed when dietary pattern moved toward a high‐salt–high‐sugar diet and Western‐mixed diet, the DII increased. Furthermore, PMS symptoms became more severe with the consumption of a high‐salt–high‐sugar diet and Western‐mixed diet.

## AUTHOR CONTRIBUTIONS

**Samaneh Farpour:** Data curation (equal); writing – original draft (equal). **Davood Soleimani:** Formal analysis (equal); writing – review and editing (equal). **Mehdi Moradinazar:** Formal analysis (equal); writing – review and editing (supporting). **Mehnoosh Samadi:** Investigation (lead); methodology (lead); project administration (lead); supervision (lead); writing – review and editing (lead).

## CONFLICT OF INTEREST STATEMENT

All authors declare that they have no conflicts of interest.

## ETHICS STATEMENT

This study was approved by the Ethics Committee of Kermanshah University of Medical Sciences (IR. KUMS. REC.1400136).

## Data Availability

The data that support the findings of this study are available on request from the corresponding author. The data are not publicly available due to privacy or ethical restrictions.
